# The clinical use of blood-test factors for Alzheimer’s disease: improving the prediction of cerebral amyloid deposition by the *QPLEX*^*TM*^*Alz plus assay kit*

**DOI:** 10.1038/s12276-021-00638-3

**Published:** 2021-06-09

**Authors:** Haeng Jun Kim, Jong-Chan Park, Keum Sim Jung, Jiyeong Kim, Ji Sung Jang, Sunghoon Kwon, Min Soo Byun, Dahyun Yi, Gihwan Byeon, Gijung Jung, Yu Kyeong Kim, Dong Young Lee, Sun-Ho Han, Inhee Mook-Jung

**Affiliations:** 1 Department of Biochemistry and Biomedical Sciences, College of Medicine, Seoul National University, Seoul, 03080 Republic of Korea; 2 SNU Dementia Research Center, College of Medicine, Seoul National University, Seoul, 03080 Republic of Korea; 3 Neuroscience Research Institute, Medical Research Center, College of Medicine, Seoul National University, Seoul, 03080 Republic of Korea; 4 Department of Neurodegenerative Disease, UCL Queen Square Institute of Neurology, University College London, London, WC1E 6BT UK; 5 QuantaMatrix Inc, Seoul, 03080 Republic of Korea; 6 Department of Neuropsychiatry, Seoul National University Bundang Hospital, Seongnam, 13620 Republic of Korea; 7 Department of Neuropsychiatry, Seoul National University Hospital, Seoul, 03080 Republic of Korea; 8 Department of Nuclear Medicine, SMG-SNU Boramae Medical Center, Seoul, 07061 Republic of Korea; 9 Department of Psychiatry, College of Medicine, Seoul National University, Seoul, 03080 Republic of Korea; 10 Institute of Human Behavioral Medicine, Medical Research Center, Seoul National University, Seoul, 03080 Korea

**Keywords:** Diagnostic markers, Alzheimer's disease, Predictive markers

## Abstract

Alzheimer’s disease (AD) is the leading cause of dementia, and many studies have focused on finding effective blood biomarkers for the accurate diagnosis of this disease. Predicting cerebral amyloid deposition is considered the key for AD diagnosis because a cerebral amyloid deposition is the hallmark of AD pathogenesis. Previously, blood biomarkers were discovered to predict cerebral amyloid deposition, and further efforts have been made to increase their sensitivity and specificity. In this study, we analyzed blood-test factors (BTFs) that can be commonly measured in medical health check-ups from 149 participants with cognitively normal, 87 patients with mild cognitive impairment, and 64 patients with clinically diagnosed AD dementia with brain amyloid imaging data available. We demonstrated that four factors among regular health check-up blood tests, cortisol, triglyceride/high-density lipoprotein cholesterol ratio, alanine aminotransferase, and free triiodothyronine, showed either a significant difference by or correlation with cerebral amyloid deposition. Furthermore, we made a prediction model for Pittsburgh compound B-positron emission tomography positivity, using BTFs and the previously discovered blood biomarkers, the *QPLEX*^*TM*^ *Alz plus assay kit* biomarker panel, and the area under the curve was significantly increased up to 0.845% with 69.4% sensitivity and 90.6% specificity. These results show that BTFs could be used as co-biomarkers and that a highly advanced prediction model for amyloid plaque deposition could be achieved by the combinational use of diverse biomarkers.

## Alzheimer’s disease: routine blood-test results could assist early diagnosis

Four blood components measured in routine tests may highlight the cerebral protein build-up indicative of Alzheimer’s disease and could improve diagnosis. Amyloid protein plaque accumulation in the brain is a hallmark of Alzheimer’s, and early detection prior to symptoms appearing is invaluable. PET imaging is currently used for detecting plaque deposition, but scientists are developing simpler detection methods using blood biomarkers such as amyloid peptide levels. Haeng Jun Kim at Seoul National University, South Korea, and co-workers demonstrated that biomarker test accuracy could be boosted by including routine blood-test data. They correlated PET imaging data with blood results from patients with mild cognitive impairment, Alzheimer’s, and healthy controls. They identified components whose levels are associated with amyloid deposition, including a liver enzyme and the hormone cortisol, and developed a proof-of-concept plaque prediction model.

## Introduction

Alzheimer’s disease (AD) is the leading cause of dementia (DEM) and the most prevalent age-related neurodegenerative disease. Pathological initiation occurs much earlier than symptoms such as cognitive impairment appear^1^. Senile plaques, composed of amyloid-beta (Aβ), have been the representative hallmark of AD pathology^2^. Aβ peptides form senile plaques through the process of oligomer and fibril formation^3^. Excess Aβ peptides and the accumulation of these peptides lead to synaptic dysfunction and neuronal death that cause cognitive dysfunction^4^. As cerebral Aβ deposition starts to occur long before the onset of clinical symptoms^5^, the early detection of cerebral Aβ deposition can realize early diagnosis of AD.

To date, the most definitive way to detect cerebral Aβ deposition has been positron emission tomography (PET) imaging using amyloid-binding radioactive molecules^6^. Pittsburgh compound B (PiB) is one of the most well-known Aβ-PET agents, and PiB-PET is a powerful tool for AD diagnosis because PiB binds specifically to Aβ^7^. However, PiB-PET imaging is unapproachable because of its high cost, concern for radiation exposure, and time-consuming process^8,9^.

To meet the need for convenient detection, many efforts have been made to find blood biomarkers to predict cerebral amyloid deposition and some have been demonstrated to show effective performance^10,11^. In particular, blood Aβ levels were demonstrated to reflect cerebral amyloid deposition effectively when quantified using special preparations with a mixture of protease inhibitors and phosphatase inhibitors (MPPs)^12^. The Aβ42 and Aβ42/40 ratios stabilized by MPPs were significantly different between the PiB– and PiB+ groups and showed a significant correlation with cerebral amyloid deposition levels. In addition, effective blood biomarkers that predict cerebral amyloid deposition were discovered and developed into a diagnostic kit that showed efficient performance in clinical trials with hundreds of AD patients^13^. Along with these discovered blood biomarkers for AD, combinational use of co-biomarkers would be helpful and increase the sensitivity and specificity of diagnostic performance if co-biomarkers could be easily monitored and exist regularly. In this study, we sought to predict cerebral Aβ deposition with routine blood-test factors (BTFs). A total of 300 individuals, comprising the experimental group, were subdivided into cognitive normal (CN), mild cognitive impairment (MCI), and AD DEM groups. We analyzed all the BTFs in routine health check-up blood tests from participants and correlated these data with PiB-PET imaging data. We selected BTFs that were significantly correlated with cerebral amyloid deposition and constructed a predictive model for cerebral amyloid deposition using these BTFs. In addition, we utilized BTF as a co-biomarker along with a previously discovered blood biomarker kit for the prediction of cerebral amyloid deposition, the *QPLEX*^*TM*^ *Alz plus assay kit*, and demonstrated significantly improved diagnostic performance^13^, which proved the possibility of BTFs as co-biomarker for the prediction of cerebral amyloid deposition.

## Materials and methods

### Recruitment of participants and neuroimaging

Participants were recruited as part of the Korean Brain Aging Study for the Early diagnosis and prediction of Alzheimer’s disease (KBASE) project. One hundred forty-nine participants with cognitively normal (CN), 87 patients with MCI, and 64 patients with clinically diagnosed AD DEM were included in this study (a total of 300 participants). They all underwent PiB-PET neuroimaging through a 3.0 T positron emission tomography–magnetic resonance imaging scanner. A total of 555 MBq of [^11^C]PiB (range, 450–610 MBq) was intravenously administered. To calculate the degree of cerebral amyloid deposition (PiB retention), four regions of interest (ROIs) were defined by an automatic anatomic algorithm and a region-combining method as follows: frontal, lateral parietal, posterior cingulate-precuneus, and lateral temporal regions. The standardized uptake value ratio (SUVR) of each ROI was a criterion for PiB-PET positivity (PiB+, >1.4 in at least one of four ROIs; PiB–, <1.4 all four ROIs)^14^. Furthermore, all participants were given comprehensive clinical and neuropsychological assessments according to the KBASE baseline assessment protocol. Detailed information on the methodology and baseline sample characteristics as described in our previous paper^15^.

### Blood sampling and storage

All participants underwent blood sampling and comprehensive laboratory blood tests. In brief, fasting blood samples were collected at 9:00 am and stored in serum separator tubes (Becton, Dickinson, and Co., Franklin Lakes, NJ, USA) or K2 EDTA tubes (BD Vacutainer Systems, Plymouth, UK). The serum tubes were centrifuged at 1300 × *g* for 10 min at room temperature (RT), and serum supernatants were collected and stored at −80 °C. The EDTA tubes were centrifuged at 700 × *g* for 5 min at RT, and plasma supernatants were gathered and stored at −80 °C. The buffy coat was also separated from the EDTA tubes for flow cytometry. Blood tests were performed at Seoul Clinical Laboratories (SCL). The details of all blood-test items (52 kinds) are shown in Table S2.

### Ethical approval

All participants in this study or their legal representatives provided informed consent to participate in this project. This study was approved by the Seoul National University Hospital Institutional Review Board.

### QPLEX^TM^ Alz plus assay (QPLEX^TM^)

Quantamatrix’s multiplex diagnostics platform *(QPLEX*^*TM*^; Quantamatrix Inc., Republic of Korea) was applied as previously described^16^ for the *QPLEX*^*TM*^ kit with microdisk technology to analyze multiplexes in a single well^17^. This system utilizes graphically coded beads that can expose antigens. In brief, diluted human plasma samples were incubated with the coded beads and antibodies in a 96-well plate. A 96-well plate was incubated in a shaking incubator at 1000 rpm for 90 min at RT. Coded beads, including immunocomplexes, were washed on a Biotek-510 magnetic wash station (Biotek, VT, USA). Fifty microliters of diluted R-phycoerythrin-conjugated streptavidin were added to each well and incubated for 15 min at RT. After incubation, the immunocomplexes were washed three times. Complexes were resuspended in 100 μl of washing buffer and analyzed.

### Statistical analyses

MedCalc 17.2 (MedCalc Software, Ostend, Belgium) and GraphPad Prism 8 (GraphPad Software, San Diego, CA, USA) were used for the data analyses. To compare the PiB– and PiB+, an independent *t* test was performed. Correlations were validated using Pearson’s correlation analysis. For the independent relationship between the factors and cerebral amyloid deposition, multiple regression was conducted with several variables. In addition, logistic regression analyses were followed by receiver operating characteristic (ROC) curve analyses. The basic formula for the algorithm applied in this study was based on a previous paper^16^. To check the multicollinearity, variance inflation factors were confirmed. Outliers were excluded from the cohort according to Grubb’s double-side outlier test (*p* < 0.05). The narrowing down of target BTFs was performed as shown in Fig. S1.

## Results

### Demographics and experimental design

**Fig. 1:**
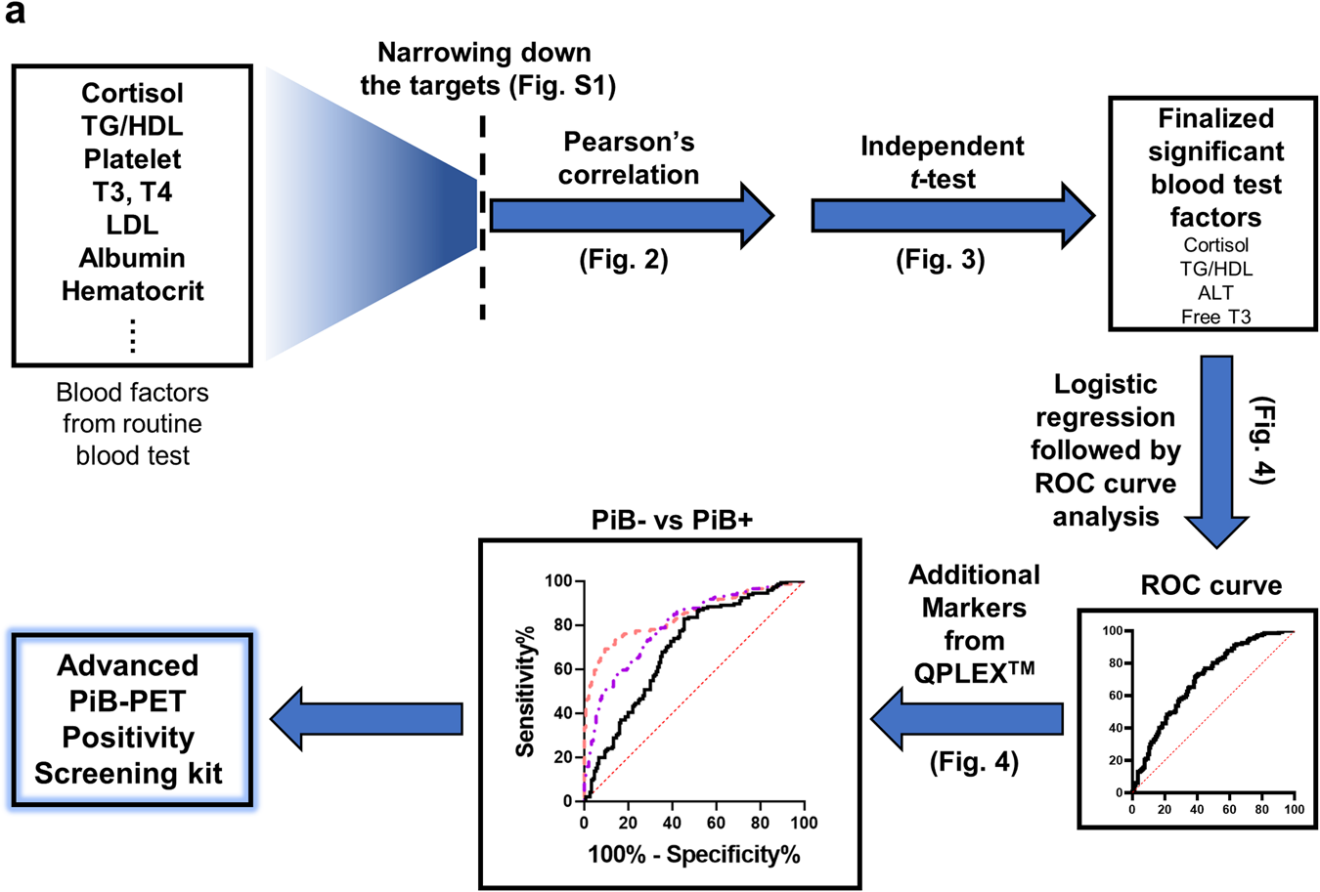
Experimental design. **a** The process of selecting a blood-test factor (BTF) candidate is simplified in the figure. First, BTFs were selected by a narrowing-down strategy (details of the narrowing-down strategy are shown in Figure S1). The BTFs then underwent Pearson’s correlation analysis and unpaired *t* test to select significant BTFs. Using the finalized BTFs, a receiver operating characteristic (ROC) curve was created by selected BTFs. Then, an advanced PiB-PET positivity screening kit was constructed by adding blood protein markers from a *QPLEX*^*TM*^ *Alz plus assay kit*.

**Fig. 2:**
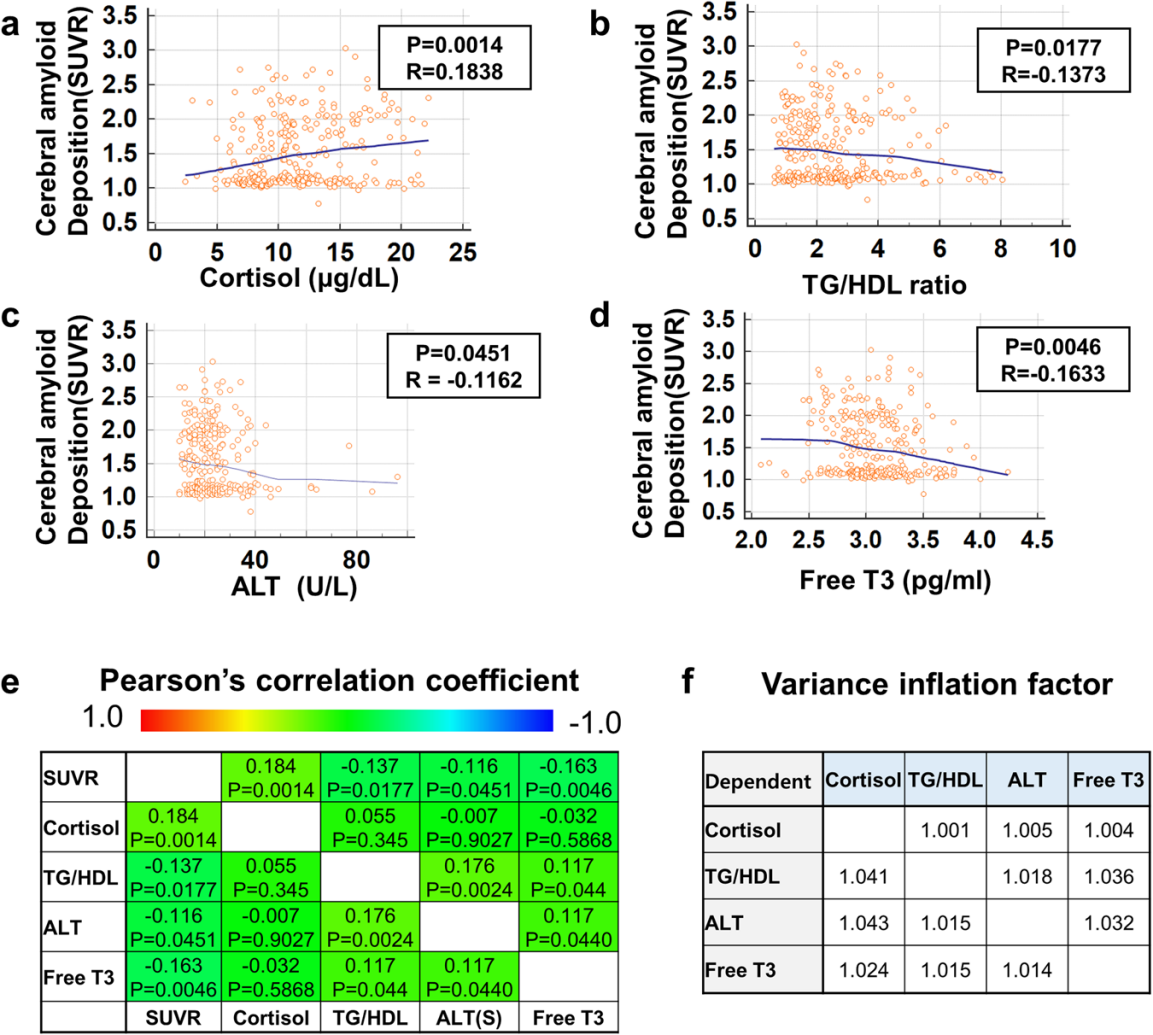
List of BTF markers shows a correlation with cerebral amyloid deposition. **a**–**d** Pearson’s correlation analysis of the indicated blood-test factors and global PiB-PET SUVR. **e** Correlogram of blood factor markers and PiB-PET SUVR. *P* values and coefficients (*R*) are shown in the box for each graph. *P* values were obtained from Pearson’s correlation analysis. **f** Variance inflation factor (VIF) was calculated from each blood-test factor. As every VIF for each factor is lower than 10, selected BTFs do not have multicollinearity. *SUVR* standard uptake value ratio, *TG/HDL* triglyceride to high-density lipoprotein ratio.

**Fig. 3:**
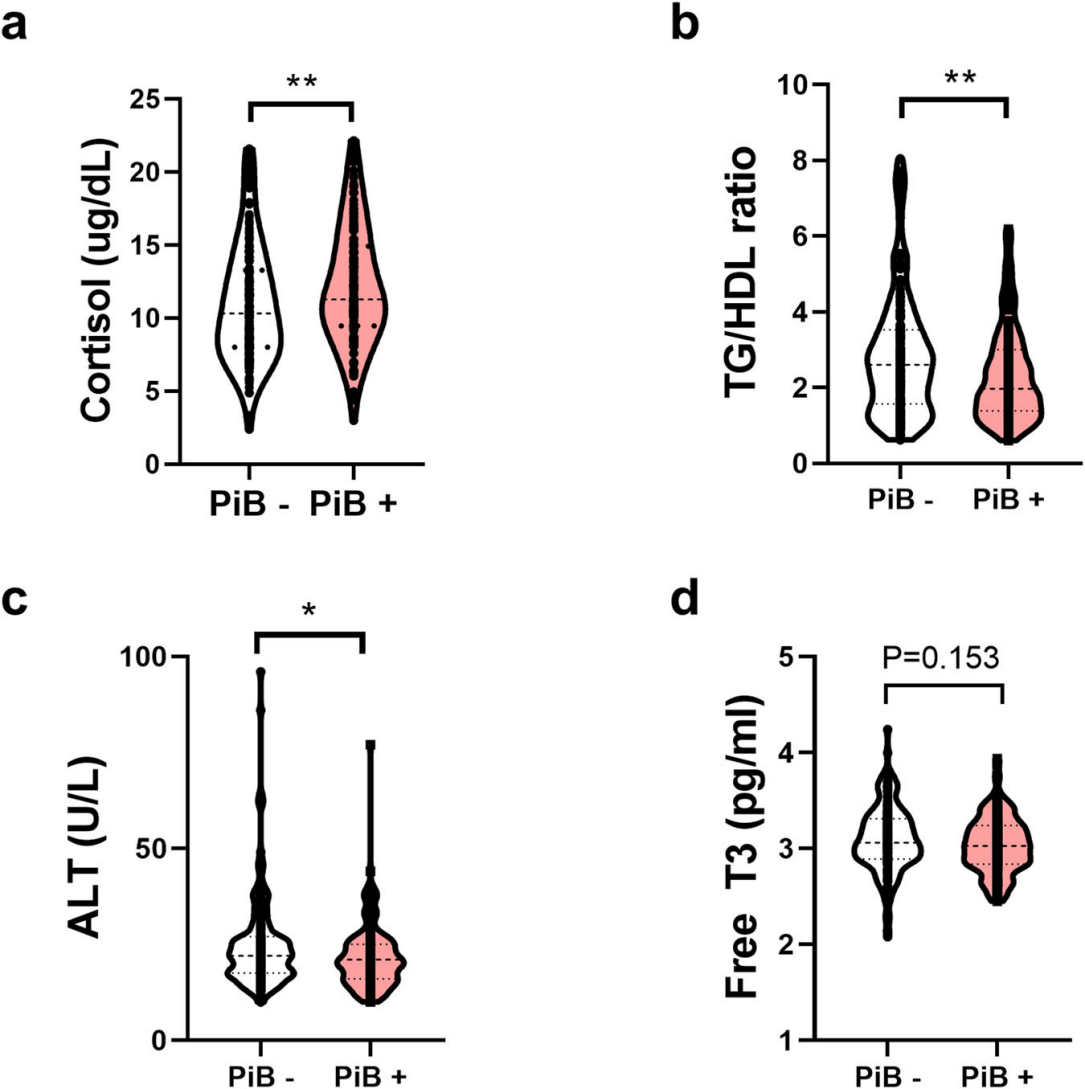
The levels of selected BTFs and comparison of PiB− and PiB+. **a**–**d** The indicated blood factors showed significant differences between the PiB-positive and PiB-negative groups. **a** Cortisol shows a significantly higher concentration in the PiB-positive group. **b**–**d** The TG/HDL ratio and ALT and free T3 concentrations were lower in the PiB-positive group. *p < 0.05, **p < 0.01 by an unpaired *t* test. *TG/HDL* triglyceride to high-density lipoprotein ratio, *ALT* alanine aminotransferase, *free T3* free triiodothyronine.

**Fig. 4:**
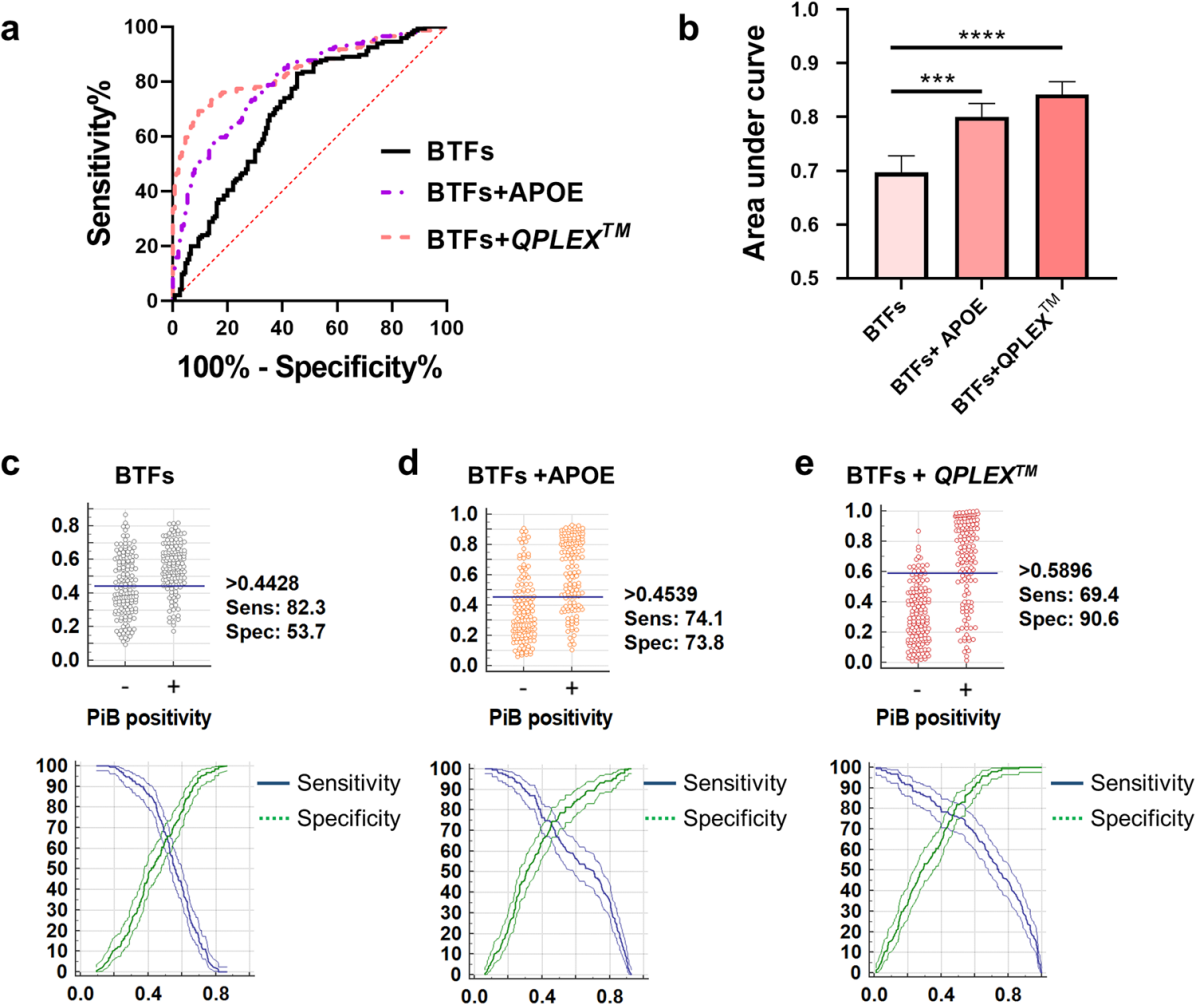
Adding the *QPLEX*^*TM*^ and the APOE genotype to the logistic regression model increased the discrimination power of the ROC curve. **a**, **b** ROC curve analysis followed by logistic regression analysis and a comparison of ROC curves. The AUC of the ROC curve was increased by adding APOE and *QPLEX*^*TM*^ biomarkers to the logistic regression model of BTFs. ***p < 0.005, ****p < 0.0001. *P* values were obtained from the comparison of ROC curve analysis results. **c**–**e** Interactive dot diagram (upper panel) and plot versus criterion value graphs (lower panel) for the indicated markers. The blue horizontal line on the interactive dot diagram represents the Youden index cutoff of each marker. *BTFs* blood-test factors, *QPLEX*^*TM*^ *QPLEX*^*TM*^ *Alz plus assay*; *APOE* the genotype of APOE.

Participants were classified as PiB– (PiB-PET negative, *n* = 150) and PiB+ (PiB-PET positive, *n* = 150) groups. The demographic details are shown in Table 1. The PiB+ group had significantly higher cerebral amyloid deposition (SUVR) than the PiB– group (PiB+, 1.92 ± 0.03; PiB–, 1.11 ± 0.01). All comparative analyses were conducted for the comparison between PiB– and PiB+. Our experimental design is shown in Fig. 1. First, to narrow down the final targets from the list of all BTFs (52 kinds from medical check-ups), the following BTFs were excluded: (i) those with *p* > 0.2 in Pearson’s correlation test; (ii) abundant proteins such as albumin; (iii) blood cell numbers (because they are not values from plasma or serum); (iv) those with problems of multicollinearity; (v) sex hormones, as they could greatly vary between men and women; and (vi) those with *p* > 0.1 in Pearson’s correlation test, even after the exclusion of outliers. Finally, four candidate BTFs remained (cortisol, triglyceride/high-density lipoprotein cholesterol ratio (TG/HDL), alanine aminotransferase (ALT), and free T3) (Fig. S1). These four candidates were finally tested by Pearson’s correlation analysis, independent *t* test, and logistic regression followed by ROC curve analysis (Figs. 2–4). Furthermore, these candidates were statistically combined with our previously identified biomarker assay panel (*QPLEX*^*TM*^ *Alz plus assay*), including four plasma protein markers^13^, galectin-3 binding protein (LGALS3BP), beta-amyloid 1–40 (Aβ1–40), angiotensin-converting enzyme, and periostin (POSTN), to improve the discrimination power for the comparison of PiB– and PiB+ (Fig. 4).

**Table 1:** Demographic data of the participants (*n* = 300).

Characteristics	PiB– (150)	PiB+ (150)	*P* value
Sex, M/F (*n*)	58/92	55/95	*P* = 0.7212^†^
Age, years, mean ± SEM	68.95 ± 0.68	73.16 ± 0.57	<0.001*
Education, mean ± SEM	10.73 ± 0.40	10.44 ± 0.39	*P* = 0.6081*
MMSE raw score, mean ± SEM	25.78 ± 0.28	21.05 ± 0.43	<0.001*
MMSE *z* score, mean ± SEM	−0.048 ± 0.09	−1.72 ± 0.15	<0.001*
CDR (*n*)	0.14 ± 0.02	0.52 ± 0.03	<0.001*
CN/MCI/dementia	113/31/6	36/56/58	<0.001^†^
PiB (SUVR), mean ± SEM	1.11 ± 0.01	1.92 ± 0.03	<0.001*
ApoE4 positivity, ε4 + /*N* (%)	22/150 (14.7%)	85/150 (56.7%)	<0.001^†^
Cortisol (μg/dl), mean ± SEM	11.00 ± 0.33	12.23 ± 0.32	<0.01*
TG/HDL ratio, mean ± SEM	2.83 ± 0.13	2.33 ± 0.10	<0.005*
ALT (U/l) ± SEM	24.96 ± 1.00	21.82 ± 0.69	<0.05*
Free T3 (pg/ml) ± SEM	3.09 ± 0.03	3.04 ± 0.02	*P* = 0.153*

*CN* cognitively normal, *MCI* mild cognitive impairment, *ADD* Alzheimer’s disease dementia, *PiB* Pittsburgh compound B, *− or +* PiB positivity, *SEM* standard error of the mean, *n* number of participants, *MMSE* mini-mental state examination, *MMSE* z score a revised value of the MMSE score with consideration for age sex and education level, *CDR* Clinical Dementia Rating, *ApoE* Apolipoprotein E, *SUVR* standardized uptake value ratio, *N* total number of participants. - *significance by *t* test, ^†^significance by chi-squared test.

### Correlation of selected BTFs with cerebral amyloid deposition

The four selected BTFs were significantly correlated with cerebral amyloid deposition (SUVR) (Fig. 2a–d). Several BTFs showed significant correlations with each other (TG/HDL and ALT, Free T3 and ALT, Free T3 and TG/HDL), but there were no problems of multicollinearity (variance inflation factor <10) (Fig. 2e–f). In addition, the levels of BTFs were significantly different between PiB– and PiB+, but free T3 showed a trend toward significance only (Fig. 3a–d). Among these four BTFs, cortisol had the highest relationship with brain amyloid deposition according to the multiple regression analyses (Table S1). These results showed that our four BTFs are associated with brain Aβ deposition, but stressful conditions of participants could play an important role in their progression of AD pathology.

### Discrimination power of BTFs on PiB-PET positivity with the combination of QPLEX^TM^ biomarkers

To verify the usefulness of the BTFs, logistic regression and ROC curve analyses were conducted (Fig. 4). The details of the ROC curve analysis are shown in Table 2. The first model containing BTFs only had highly significant *p* values (*p* < 0.0001, Table 2). Next, we added ApoE genotype or *QPLEX*^*TM*^ biomarkers as additional variables, and then three logistic regression models for ROC curve analysis were generated as follows: (i) BTFs, (ii) BTFs + ApoE, and (iii) BTFs + *QPLEX*^*TM*^ (Fig. 4a). Age and sex were used as covariates. The second and third models (BTFs + ApoE, BTFs + *QPLEX*^*TM*^) showed a higher area under the curve (AUC) than the first model (BTFs) (BTFs + ApoE, 0.797 with 74.1% sensitivity and 73.8% specificity; BTFs + *QPLEX*^*TM*^, 0.845 with 69.4% sensitivity and 90.6% specificity; Fig. 4b and Table 2). An interactive dot diagram showed the Youden index cutoff criteria of the three models (Fig. 4c–e, upper panel). The plot versus criterion value graphs representing the tendency of sensitivity and specificity are also shown (Fig. 4c–e, lower panel). These results showed that the BTFs improved the discrimination power of our previous *QPLEX*^*TM*^ biomarker panel, from an AUC of 0.834 to an AUC of 0.845^13^ (*P* = 0.00383 by net reclassification improvement method^18^), and can be used for the achievement of advanced PiB-PET positivity screening tools using human blood (Fig. S2).

**Table 2:** Detailed information of ROC curve analysis.

PiB- vs PiB+	AUC	Sensitivity (%)	Specificity (%)	*P* value	Cutoff criterion	95% CI of AUC	z-statistic
BTFs	0.694	82.31	53.69	<0.0001	>0.443	0.638–0.746	6.316
BTFs + APOE geno	0.797	74.15	73.83	<0.0001	>0.453	0.764–0.841	11.534
BTFs + QPLEX^TM^	0.845	69.39	90.60	<0.0001	>0.599	0.799–0.884	14.805

PiB−, *n* = 150; PiB+, *n* = 150.

*AUC* area under the curve, *BTFs* blood-test factors, *ApoE* apolipoprotein E genotype, *SUVR* standardized uptake value ratio.

Sensitivity and specificity were selected by the Youden index.

## Discussion

BTFs in routine health check-ups are easily accessible indicators for monitoring the general health condition, nutrition balance, and each organ’s function. Some BTFs are also used to monitor the pathological initiation, progression, and severity of disease, and certain BTFs even indicate unusual events in the brain beyond the blood-brain barrier (BBB). For instance, plasma homocysteine levels are associated with increased risk for cognitive decline, DEM, and AD^19–21^. Interestingly, plasma phosphorus levels exhibited a significant correlation with cerebral Aβ deposition in cognitively impaired individuals^11^. In this study, we investigated the BTFs of a total of 300 individuals of CN, MCI, and AD patients who underwent PiB-PET scanning (150 PiB– and 150 PiB+). Fifty-two BTFs were analyzed from these 300 individuals, and four BTFs, cortisol, TG/HDL, ALT, and free T3, were selected as effective biomarkers to predict brain Aβ deposition. These four BTFs showed a significant correlation with cerebral amyloid deposition in the *t* test and Pearson’s correlation. To improve the effectiveness of these BTFs as biomarkers, genetic markers for AD and previously discovered blood biomarkers for cerebral amyloid deposition were integrated. Integrated models with combinational use of biomarkers showed high predictability for cerebral amyloid deposition.

Some hormonal levels have long been discussed for their possible connection to AD pathogenesis. However, no evidence of an exact link between blood hormone levels and brain Aβ deposition has been suggested. Cortisol and free T3 are examples of those cases.

Corticosteroids are known to be associated with brain function, including mood, stress, anxiety, and cognition^22,23^. Corticosteroids, especially cortisol in humans, cross the BBB easily and affect cognitive function by binding to intracellular receptors, such as glucocorticoid receptors and mineralocorticoid receptors, in particular, brain regions associated with cognitive function^24^. In addition, it has been suggested that elevated cortisol levels are associated with cognitive impairment and AD pathology^25^. Furthermore, longitudinal detection of urinary cortisol levels predicts the risk for AD^26^. One report shows that high plasma cortisol levels accelerate the effect of brain Aβ deposition on cognitive decline in cognitively healthy adults^27^. However, the relationship between blood cortisol levels and brain Aβ pathological changes is not known.

Thyroid hormones have long been investigated in relation to cognitive dysfunction and involvement with AD pathology. Thyroid levels showed a tight relationship with cognitive impairment in subcortical ischemic vascular disease, showing decreased serum total triiodothyronine, free T3, and increased thyroid-stimulating hormone (TSH) levels^28^. Serum-free thyroxine (T4) levels were found to be associated with cerebral Aβ deposition in CN individuals, whereas serum TSH levels showed a significant negative correlation with glucose metabolism in the precuneus/posterior cingulate cortex in CN individuals^29^. A significant positive correlation was found between TSH levels and cortical glucose consumption detected by 2-deoxy-2-(F-18) fluoro-D-glucose (F-18 FDG) PET, but no significant relation was found between glucose consumption and serum T3 and T4 levels in the AD population^30^. No definitive correlations have been discovered between blood-free T3 levels and brain Aβ deposition.

The case of blood TG/HDL levels has been similar to that above. No definitive correlations have been discovered between blood TG/HDL levels and brain Aβ deposition, even though abnormalities in lipid metabolism and cholesterol levels have long been proposed as important mechanisms related to AD pathogenesis^31^. Previously, apolipoprotein AI, the major component of HDL, showed decreased plasma levels in AD and DEM patients compared to normal control^32^. In addition, higher TG levels at midlife were related to changes in CSF Aβ42 levels and predicted brain Aβ deposition and tau pathology later in CN individuals^21^.

In contrast to cortisol and free T3, not much is known or has been investigated about a possible relationship between ALT and AD. Previously, a study reported no significant differences in glutamic pyruvate transaminase, also called ALT, activities in four different regions of the brain from normal and AD patients^33^, whereas another study suggested liver dysfunction in AD patients, showing lower albumin and a higher AST/ALT ratio^34^. However, they failed to show a significant decrease in ALT in AD patients even though the mean value of ALT tended to decrease in AD patients compared to normal controls. Recently, a report showed that serum ALT levels are associated with cognitive performance, brain glucose metabolism, and CSF Aβ42 levels in AD. However, the authors suggested that there was no significant correlation between ALT levels and global cortical amyloid deposition^35^. Interestingly, in our study, a significant decrease in peripheral ALT levels was demonstrated in PiB+ individuals (Fig. 3), and a significant correlation between ALT levels and global amyloid deposition was demonstrated (Fig. 2c, *p* = 0.0451). We utilized PiB-PET imaging to measure cerebral amyloid deposition, while the other study used florbetapir as an imaging tracer, which might be a possible explanation for the conflicting results. Although PiB-PET and florbetapir PET images are strongly associated^36^, a recent study proposed that differences were observed after Centiloid scale conversion and that florbetapir exhibited higher variability than PiB^37^. In our study, plasma AST levels showed no difference between PiB– and PiB+ (data not shown), which is consistent with other previous studies showing no difference in AST levels in AD patients^34^. In addition, plasma ALT levels were significantly correlated with the levels of other cerebral Aβ-related BTFs in our study, such as TG/HDL and free T3, without multicollinearity (Fig. 2), which reinforces the correlation between ALT and cerebral amyloid deposition.

The combined use of multiple biomarkers is the ideal strategy in AD diagnosis. The combination of cognitive markers, CSF protein biomarkers, and imaging biomarkers significantly improved the accuracy of predicting conversion from MCI to AD^38,39^. A gradual increase in statistical power was shown by increasing the number of biomarkers used in identifying MCI patients susceptible to AD^40^. Furthermore, the combinational use of various blood biomarkers increased the power of diagnostic accuracy to predict brain Aβ deposition^41–43^.

We tested whether our BTFs could be synergistically valuable for predicting brain Aβ deposition to previously discovered blood biomarkers or genetic markers for AD. The ApoE4 genotype is the most well-known risk factor for sporadic AD^44^. In addition, recently discovered blood biomarkers for predicting brain Aβ deposition, plasma levels of LGALS3BP, ACE, periostin, and Aβ 40, exhibited incremental predictive power by combinational use one by one^42^, and the subsequent development of the *QPLEX*^*TM*^ *Alz plus assay kit*, composed of these multiple biomarkers, reinforced the improved diagnostic accuracy for combinational use of biomarkers^13^. Combinational use of a BTF with either ApoE or *QPLEX*^*TM*^ biomarkers demonstrated a significant increase in the AUC, showing an AUCs of 0.797 for BTFs+APOE and 0.845 for BTFs+*QPLEX*^*TM*^ (Fig. 4*p* < 0.001, *p* < 0.0001, respectively), compared with a BTF only (AUC 0.694), APOE only (AUC 0.781), and *QPLEX*^*TM*^ only (AUC 0.839). These results show that BTFs could be a useful co-biomarkers for combinational use with other blood biomarkers for AD diagnosis. In addition, the usage of BTFs as co-biomarkers could be further expanded to combinational use with other biomarkers, including CSF biomarkers, cognitive biomarkers, or PET imaging biomarkers, to improve accuracy and efficiency. Furthermore, we conducted logistic regression to distinguish the stage of AD and obtained AUCs by using BTFs and BTFs+QPLEX (Table S3). Although ROC curve models did not have higher AUCs than the PiB-PET positivity models, they were significant in discriminating against CN from other diagnosed states (MCI and AD). As expected, adding QPLEX to BTFs increased the AUC and the significance of the ROC curves. The relatively lower discrimination power for diagnosis is because we determined BTFs and QPLEX factors for PiB-PET positivity. The relatively lower discrimination power for diagnosis is because we determined BTFs and QPLEX factors for PiB-PET positivity. In future studies, the combinational use of diverse biomarkers with BTFs could be tested to maximize the efficacy of diagnosis for brain Aβ deposition. In addition, a longitudinal study will be necessary to monitor the change in BTF levels depending on the process of disease progression.

## Supplementary information

Supplementary information
