# Supplementary material for: The clinical use of blood-test factors for Alzheimer’s disease: improving the prediction of cerebral amyloid deposition by the QPLEXTMAlz plus assay kit
Source: Exp Mol Med. 2021 Jun 9;53(6):1046–54. doi: 10.1038/s12276-021-00638-3 (PMC8257730; doi:10.1038/s12276-021-00638-3)
Supplement: Supplementary file 1 — Supplementary information [file 12276_2021_638_MOESM1_ESM.docx]

| BTFs + Sex + Age | | | | | | | | | | | | | |
| --- | --- | --- | --- | --- | --- | --- | --- | --- | --- | --- | --- | --- | --- |
| Dependent Y | | **Cerebral amyloid deposition (SUVR)** | | | | | | | | | | | |
| Sample size | | | | | | | | | | | | 296 | |
| Coefficient of determination R^2^ | | | | | | | | | | | | 0.1090 | |
| R^2^-adjusted | | | | | | | | | | | | 0.09047 | |
| Multiple correlation coefficient | | | | | | | | | | | | 0.3301 | |
| Residual standard deviation | | | | | | | | | | | | 0.4645 | |
| Ind. variables | Coefficient | | Std. Error | | | | t | P | | | r_partial_ | r_semipartial_ | VIF |
| (Constant) | 1.12341 | |  | | |  | |  | | |  |  |  |
| Cortisol | 0.02306 | | 0.006851 | | | 3.366 | | 0.0009 | | | 0.1942 | 0.1869 | 1.034 |
| ALT(S) | -0.002471 | | 0.002625 | | | -0.942 | | 0.3472 | | | -0.05530 | 0.05228 | 1.068 |
| Free T3 | -0.1459 | | 0.08859 | | | -1.647 | | 0.1007 | | | -0.09640 | 0.09143 | 1.102 |
| TG/HDL | -0.03713 | | 0.01889 | | | -1.966 | | 0.0503 | | | -0.1149 | 0.1091 | 1.053 |
| Age | 0.007354 | | 0.003580 | | | 2.054 | | 0.0409 | | | 0.1200 | 0.1141 | 1.104 |
| Sex | 0.1369 | | 0.05727 | | | 2.390 | | 0.0175 | | | 0.1392 | 0.1327 | 1.051 |
| BTFs + APOE genotype + Sex + Age | | | | | | | | | | | | | |
| Dependent Y | **Cerebral amyloid deposition (SUVR)** | | | | | | | | | | | | |
| Sample size | | | | | | | | | | | | 296 | |
| Coefficient of determination R^2^ | | | | | | | | | | | | 0.2676 | |
| R^2^-adjusted | | | | | | | | | | | | 0.2498 | |
| Multiple correlation coefficient | | | | | | | | | | | | 0.5173 | |
| Residual standard deviation | | | | | | | | | | | | 0.4219 | |
| Ind. variables | **Coefficient** | | **Std. Error** | | **t** | | | **P** | | **r_partial_** | | **r_semipartial_** | **VIF** |
| (Constant) | 1.2012 | |  | |  | | |  | |  | |  |  |
| Cortisol | 0.01538 | | 0.006297 | | 2.442 | | | 0.0152 | | 0.1424 | | 0.1231 | 1.059 |
| ALT(S) | -0.001832 | | 0.002385 | | -0.768 | | | 0.4431 | | -0.04521 | | 0.03873 | 1.069 |
| Free T3 | -0.1054 | | 0.08062 | | -1.308 | | | 0.1920 | | -0.07683 | | 0.06595 | 1.106 |
| TG/HDL | -0.03454 | | 0.01716 | | -2.013 | | | 0.0451 | | -0.1178 | | 0.1015 | 1.053 |
| Age | 0.005262 | | 0.003262 | | 1.613 | | | 0.1078 | | 0.09463 | | 0.08135 | 1.111 |
| Sex | 0.1015 | | 0.05221 | | 1.944 | | | 0.0528 | | 0.1138 | | 0.09804 | 1.058 |
| APOE genotype | 0.4152 | | 0.05257 | | 7.897 | | | <0.0001 | | 0.4219 | | 0.3983 | 1.052 |
| BTFs + QPLEX^TM^ markers + Sex + Age | | | | | | | | | | | | | |
| Dependent Y | | | | | | | **Cerebral amyloid deposition (SUVR)** | | | | | | |
| Sample size | | | | | | | | | | | | 296 | |
| Coefficient of determination R^2^ | | | | | | | | | | | | 0.3113 | |
| R^2^-adjusted | | | | | | | | | | | | 0.2846 | |
| Multiple correlation coefficient | | | | | | | | | | | | 0.5579 | |
| Residual standard deviation | | | | | | | | | | | | 0.4118 | |
| Ind. variables | **Coefficient** | | **Std. Error** | **t** | | | | **P** | **r_partial_** | | | **r_semipartial_** | **VIF** |
| Constant) | 1.6655 | |  |  | | | |  |  | | |  |  |
| Cortisol | 0.02435 | | 0.006463 | 3.768 | | | | 0.0002 | 0.2179 | | | 0.1959 | 1.049 |
| ALT(S) | 0.0003331 | | 0.002502 | 0.133 | | | | 0.8942 | 0.007885 | | | 0.006922 | 1.106 |
| Free T3 | -0.1398 | | 0.08338 | -1.676 | | | | 0.0948 | -0.09881 | | | 0.08717 | 1.113 |
| TG/HDL | -0.02163 | | 0.01792 | -1.207 | | | | 0.2286 | -0.07130 | | | 0.06275 | 1.081 |
| Age | 0.001900 | | 0.003506 | 0.542 | | | | 0.5882 | 0.03209 | | | 0.02819 | 1.207 |
| Sex | 0.1881 | | 0.05478 | 3.434 | | | | 0.0007 | 0.1993 | | | 0.1785 | 1.096 |
| Aβ1-40 | 0.0003777 | | 0.0002724 | 1.387 | | | | 0.1666 | 0.08187 | | | 0.07211 | 1.102 |
| ACE | -0.0004878 | | 0.0002400 | -2.033 | | | | 0.0430 | -0.1196 | | | 0.1057 | 1.309 |
| LGALS3BP | -0.00006673 | | 0.00001355 | -4.924 | | | | <0.0001 | -0.2800 | | | 0.2561 | 1.215 |
| POSTN | 0.01016 | | 0.002187 | 4.646 | | | | <0.0001 | 0.2654 | | | 0.2416 | 1.365 |

**Table S1. Multiple regression analyses**

***Abbreviations:** SUVR, standardized uptake value ratio; Ind., independent; VIF, variance inflation factor; ALT, Alanine aminotransferase; Free T3, Free triiodothyronine; TG/HDL, Triglyceride/High density lipoprotein ratio; LGALS3BP, galectin-3 binding protein; ACE, angiotensin converting enzyme; POSTN, periostin; ApoE, apolipoprotein E; BTFs, Blood test factors.

**Table S2. List of methods and information of blood tests equipment and reagents**

| **Test item** | **Test methods** | **Equipments for tests**  **(Company, country)** | **Reagents for tests**  **(Company, country)** | **Reference value for normal condition** |
| --- | --- | --- | --- | --- |
| HbA1C | HPLC | Varient II Turbo (Bio-Rad, USA) | Variant II HbA1c program(BIO-RAD, USA) | 5.02 - 6.28% |
| Glucose (FBS) | Colorimetric | ADVIA 1800 Auto Analyzer (Siemens, USA) | GLUH(Siemens,UK) | 74-106mg/dL |
| Insulin | CIA | ADVIA Centaur XP(Siemens,USA) | IRI(Siemens,JAPAN) | 2-25 mIU/L |
| T.Cholesterol | Colorimetric | ADVIA 1800 Auto Analyzer(Siemens, USA) | Cholesterol,total(Siemens,UK) | <200mg/dL |
| Trglyceride | Colorimetric | ADVIA 1800 Auto Analyzer(Siemens, USA) | Triglyceride(Siemens,UK) | <250mg/dL |
| HDL-Cholesterol | Colorimetric | ADVIA 1800 Auto Analyzer(Siemens, USA) | HDL-Cholesterol(Siemens,UK) | 40-60mg/dL |
| LDL-Cholesterol | Colorimetric | ADVIA 1800 Auto Analyzer(Siemens, USA) | LDL-Cholesterol(Siemens,UK) | <130mg/dL |
| Testosteron | CIA | ADVIA Centaur XP(Siemens,USA) | Testosteron(Siemens,USA) | Male  Age 19-71y 2.41-8.27 ng/mL  Female  15-75y 0.14-0.76 ng/mL |
| E2 | CIA | ADVIA Centaur XP(Siemens,USA) | E2(Siemens,USA) | Male  ≤39.8 pg/mL Female  Follicular phase 19.5-144.2 pg/mL Ovulation phase 63.9-356.7 pg/mL  Luteal phase 55.8-214.2 pg/mL  Menopause ≤32.2 pg/mL |
| FSH | CIA | ADVIA Centaur XP(Siemens,USA) | FSH(Siemens,USA) | Male  13-70y 1.40-18.10 mIU/mL  Female  Follicular phase 2.50-10.20 mIU/mL Ovulation phase 3.40-33.40 mIU/mL Luteal phase 1.50-9.10mIU/mL Pregnant <0.30 mIU/mL Menopause 23.00-116.30mIU/mL |
| LH | CIA | ADVIA Centaur XP(Siemens,USA) | LH(Siemens,USA) | Male  20-70y 1.50- 9.30 mIU/mL >71y 3.10-34.60 mIU/mL  Female  Follicular phase 1.90-12.50 mIU/mL Ovulation phase 8.70-76.30 mIU/mL Luteal phase 0.50-16.90 mIU/mL Pregnant <0.10- 1.50 mIU/mL Menopause 15.90-54.00 mIU/mL Contraception 0.70- 5.60 mIU/mL  Infant <0.10-6.00 mIU/mL |
| ApoE geotyping | PCR | Bio-Rad PCR machine | ApoE Genotyping PCR Kit (BioCore) | 6 types of genotype E2/E2, E2/E3, E2/E4, E3/E3, E3/E4, E4/E4 |
| Apolipoprotein A1 | Immunoturbidimetric assay | COBAS INTEGRA 800 (Roche Diagnostics) | APOAT (Roche Diagnostics | Male 110-180 Female 110-205 mg/dL |
| Apolipoprotein B | Immunoturbidimetric assay | COBAS INTEGRA 800 (Roche Diagnostics) | APOBT (Roche Diagnostics | Male 60-140 Female 50-130 mg/dL |
| Protein,total | Colorimetric | ADVIA 1800 Auto Analyzer(Siemens, USA) | T.Protein(Siemens,UK) | 5.7-8.2 g/dL |
| Albumin | Colorimetric | ADVIA 1800 Auto Analyzer(Siemens, USA) | Albumin (Siemens,UK) | 3.2-4.8 g/dL |
| BUN | Colorimetric | ADVIA 1800 Auto Analyzer(Siemens, USA) | UN(Siemens,UK) | 9.0-23.0 mg/dL |
| Creatinine | Colorimetric | ADVIA 1800 Auto Analyzer(Siemens, USA) | Creatinine (Siemens,UK) | Male 0.9-1.3 mg/dL Female 0.6-1.1 mg/dL |
| Uric acid | Colorimetric | ADVIA 1800 Auto Analyzer(Siemens, USA) | UA(Siemens,UK) | Male 3.7-9.2 mg/dL Female 3.1-7.8 mg/dL |
| Bilirubin,total | Colorimetric | ADVIA 1800 Auto Analyzer(Siemens, USA) | TBIL_2(Siemens,UK) | 0.3-1.2 mg/dL |
| AST | Colorimetric | ADVIA 1800 Auto Analyzer(Siemens, USA) | AST(Siemens,UK) | <34 U/L |
| ALT | Colorimetric | ADVIA 1800 Auto Analyzer(Siemens, USA) | ALT(Siemens,UK) | 10-49 U/L |
| Alk. phosphatase | Colorimetric | ADVIA 1800 Auto Analyzer(Siemens, USA) | ALPAMP(Siemens,UK) | Adult 45-129 U/L Growth spurt (under 15y) 117-390 U/L |
| Phosphorus | Colorimetric | ADVIA 1800 Auto Analyzer(Siemens, USA) | Pi(Siemens,UK) | 2.4-5.1 mg/dL |
| Calcium | Colorimetric | ADVIA 1800 Auto Analyzer(Siemens, USA) | Ca_2(Siemens,UK) | 8.6-10.5 mg/dL |
| Iron(Fe) | Colorimetric | ADVIA 1800 Auto Analyzer(Siemens, USA) | L-Type Fe.N(WAKO,Japan) | Male 44-192 ug/dL Female 29-164 ug/dL |
| T3 | CIA | ADVIA Centaur XP(Siemens,USA) | T3(Siemens,USA) | 1-23mo 117-239 ng/dL 2-12y 105-207 ng/dL 13-21y 86-192 ng/dL >21y 60-181 ng/dL |
| Free T3 | CIA | ADVIA Centaur XP(Siemens,USA) | Free T3(Siemens,USA) | 1-23mo 3.3-5.2 pg/mL 2-12y 3.3-4.8 pg/mL 13-21y 3.0-4.7 pg/mL >21y 2.30-4.2 pg/mL |
| TSH | CIA | ADVIA Centaur XP(Siemens,USA) | TSH(Siemens,USA) | 2-12y 0.640-6.270 uIU/mL 12-18y 0.510-4.940 uIU/mL >18y 0.550-4.780 uIU/mL |
| Free T4 | CIA | ADVIA Centaur XP(Siemens,USA) | Free T4(Siemens,USA) | 1-23mo 0.94-1.44 ng/dL 2-12y 0.86-1.40 ng/dL 13-21y 0.83-1.43 ng/dL >21y 0.89-1.76 ng/dL |
| Homocysteine | CIA | ADVIA Centaur XP(Siemens,USA) | HCY(Siemens,USA) | 5.0-13.9 umol/L |
| Cortisol | CIA | ADVIA Centaur XP(Siemens,USA) | Cortisol(Siemens,USA) | Morning 5.27-22.45 ug/dL Afternoon 3.44-16.76 ug/dL |
| ESR | Westergren | Westergren Tube VACUETTE®Single-use pipettes ESR(greiner bio-one, Germany) |  | Male <15 mm/hr Female <20 mm/hr |
| CBCs | Flow Cytometry | ADVIA 2120i (Siemens, USA) | CBC time pack (Siemens,USA) |  |
| Hemoglobin |  |  |  | M 13.3-16.7 g/dL F 11.6-14.2 g/dL |
| Hematocrit |  |  |  | 34.5-52.0 % |
| WBC |  |  |  | 3.8-10.7 Thous/uL |
| RBC |  |  |  | 3.75-5.44 Mil/uL |
| Platelet |  |  |  | 147-372 Thous/uL |
| RDW |  |  |  | 11.0-15.0% |
| PDW |  |  |  | 39.0-66.0% |
| Diff. count |  |  |  |  |
| Segment |  |  |  | 38.0-78.0% |
| Lymphocyte |  |  |  | 17.0-46.0% |
| Monocyte |  |  |  | 2.0-8.0% |
| Eosinophil |  |  |  | 1.0-6.0% |
| Basophil |  |  |  | 0.0-2.0% |
| Transferrin | Immunoturbidimetric assay | COBAS INTEGRA 800 (Roche Diagnostics | TRSF2 (Roche Diagnostics | Male 22-322 Female 10-291 |
| Ceruloplasmin | Immunoturbidimetric assay | COBAS INTEGRA 800 (Roche Diagnostics | CERU(Roche Diagnostics | Male 16.2-35.6 Female 17.9-53.3 |
| RPR | Immunoturbidimetric assay | COBAS 8000 (Roche Diagnostics) | RPR REAGENT (SEKISUI, UK) | <1.0 R P R UNIT |
| SHBG | ECLIA | Cobas e602 (Roche Diagnostics) | SHBG (Roche Diagnostics) | Male 20-49y 18.3-54.1  >50y 20.6-76.7 Female 20-49y 32.4-128.0  >50y 27.1-128.0 nmol/L |
| Ferritin | CIA | ADVIA Centaur XP(Siemens,USA) | Ferritin (Siemens,USA) | Male 22-322 Female 10-291 ng/mL |
| Vitamin B12 | Radioimmunoassay | Gamma-counter | Radioassay Kit Vitamin B12 [^57^Co] / Folate[^125^I] | 160-970 pg/mL |
| Folate | Radioimmunoassay | Gamma-counter | Radioassay Kit Vitamin B12 [^57^Co] / Folate[^125^I] | 1.5-16.9 ng/mL |
| Zinc (S) | ICP-MS | 820-MS (Bruker / Austrailia) | ① Zinc standard solution : SIGMA, U.S.A ② Nitric acid : Merck, Germany ③ Water (HPLC Grade.) : Millipore, U.S.A ④ Serum control Level 1, 2 (Clincheck, Germany) | 66.0-110.0 ug/dL |
| Copper (S) | ICP-MS | 820-MS (Bruker / Austrailia) | ① Copper standard solution : SIGMA, U.S.A ② Nitric acid : Merck, Germany ③ Water (HPLC Grade.) : Millipore, U.S.A ④ Serum control Level 1, 2 (Clincheck, Germany) | 75.0-145.0 ug/dL |

| **in PiB+ group** |  |  |  |
| --- | --- | --- | --- |
|  | Discriminating  groups | AUC of  ROC curve | P value |
| BTFs only | MCI vs CN | 0.708 | P = 0.0874 |
|  | DEM vs CN | 0.711 | P = 0.0353 |
|  | DEM vs MCI | 0.561 | P = 0.9785 |
|  | MCI+DEM vs CN | 0.71 | P = 0.0266 |
| BTFs+QPLEX | MCI vs CN | 0.74 | P = 0.1229 |
|  | DEM vs CN | 0.816 | P = 0.0006 |
|  | DEM vs MCI | 0.651 | P = 0.3459 |
|  | MCI+DEM vs CN | 0.748 | P = 0.0132 |
|  |  |  |  |
| **in Total group** |  |  |  |
|  | Discriminating  groups | AUC of  ROC curve | P value |
| BTFs only | MCI vs CN | 0.68 | P = 0.0004 |
|  | DEM vs CN | 0.686 | P = 0.0062 |
|  | DEM vs MCI | 0.614 | P = 0.7451 |
|  | MCI+DEM vs CN | 0.677 | P < 0.0001 |
| BTFs+QPLEX | MCI vs CN | 0.716 | P = 0.0006 |
|  | DEM vs CN | 0.757 | P < 0.0001 |
|  | DEM vs MCI | 0.653 | P = 0.4232 |
|  | MCI+DEM vs CN | 0.723 | P < 0.0001 |

**Table S3. AUCs of models for discriminating stages of AD**

***Abbreviations:** BTFs, Blood test factors; CN, cognitively normal; MCI, mild cognitive impairment, AD dementia, DEM.


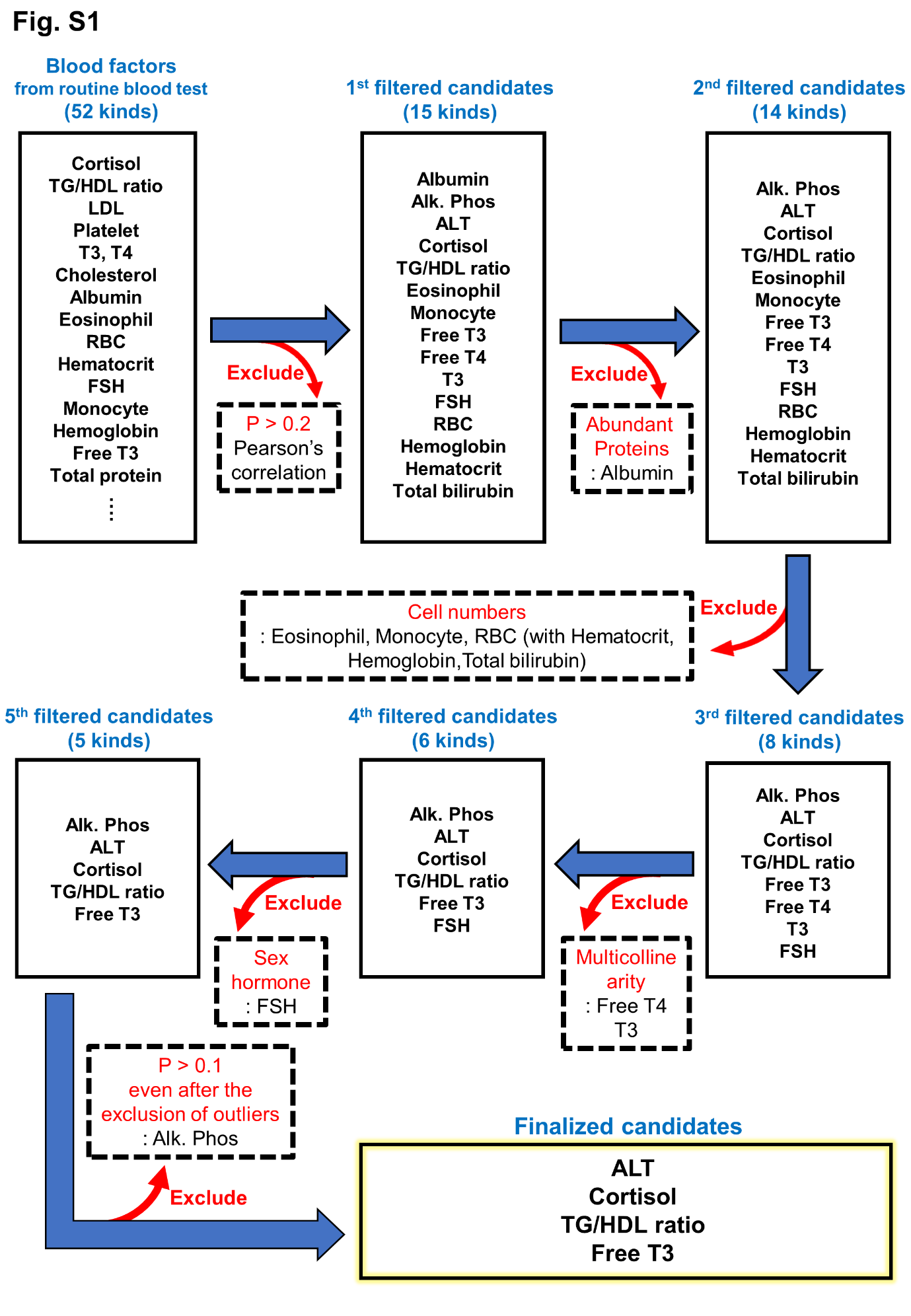


**Figure S1. Blood-test factors (BTFs) from routine blood tests were narrowed down to select targets.** To narrow down blood factors, we analyzed correlation of all blood factors from routine blood-tests between SUVR by Pearson’s correlation. BTFs are excluded if the significance of Pearson’s correlation was P > 0.2. Abundant proteins and factors representing cell number were excluded from candidates. RBC related factors (Hematocrit, Hemoglobin, Total bilirubin) are excluded with RBC because of their multicollinearity. Free T4 and total T3 are excluded because of their multicollinearity with Free T3. VIF values calculated by multiple regression of RBC related factors and Free T4 and total T3 were higher than 10. Sex hormone (FSH) is excluded to use marker for sex independent condition. Finally, even after the outliers are excluded from the Grubbs test, factors that represent P > 0.1 are excluded. SUVR, standardized uptake value ratio; Ind., independent; VIF, variance inflation factor; ALT, Alanine aminotransferase; Free T3, Free triiodothyronine; T4, thyroxine TG/HDL, Triglyceride/High-density lipoprotein ratio; LGALS3BP, galectin-3 binding protein; ACE, angiotensin-converting enzyme; POSTN, periostin; ApoE, apolipoprotein E; RBC, red blood cell; LDL, low-density lipoprotein; FSH, Follicle-stimulating hormone.


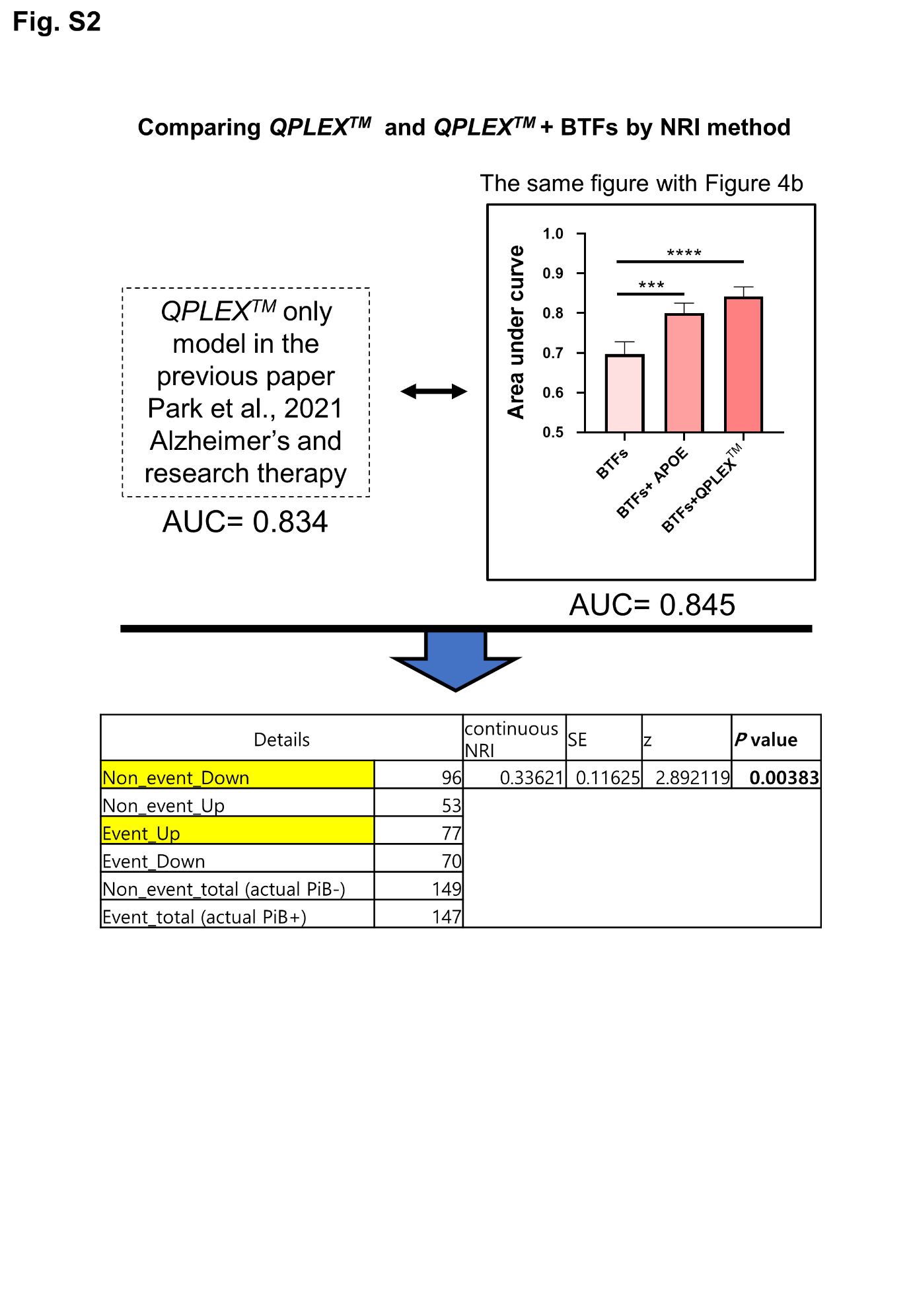


**Figure S2. Adding BTFs on *QPLEX^TM^* increased predictive ability.** To confirm an increase of predictive ability by adding BTFs as a co-biomarker, *QPLEX^TM^* only model on the previous paper was compared to *QPLEX^TM^* + BTFs model. Two models were tested by the net reclassification improvement method. To run the test, samples that have the same predictive possibility in each model were excluded. *QPLEX^TM^* + BTFs model reduced the predictive possibility of 96 PiB-PET negative samples and the increased predictive possibility of 77 PiB-PET positive samples. Total 173 of predictive possibilities were improved by adding BTFs on the *QPLEX^TM^* only model (P = 0.00383). BTFs, Blood test factors; *QPLEX^TM^*, *QPLEX^TM^ Alz plus assay*; Non-event, PiB-PET negative group; Event, PiB-PET positive group; Down, number of decreased predictive possibility; Up, number of increased predictive possibility.
